# A new research proposal to prevent hydrogen embrittlement for nuclear waste container by bacteria-a mini review

**DOI:** 10.3389/fmicb.2023.1304703

**Published:** 2023-11-23

**Authors:** Qichao Zhang, Yishan Jiang, Xin Zhao, Jizhou Duan, Luning Chen, Ying Xu

**Affiliations:** ^1^Navy Submarine Academy, Qingdao, China; ^2^CAS Key Laboratory of Marine Environment of Corrosion and Bio-fouling, Institute of Oceanology, Chinese Academy of Sciences, Qingdao, China

**Keywords:** microbial corrosion, hydrogen embrittlement, hydrogen consuming microorganisms, deep geological disposal, nuclear waste

## Abstract

A large amount of nuclear waste produced in the process of nuclear energy utilization has always been a key problem to be solved urgently for nuclear safety. At present, “deep geological disposal” is a feasible method and generally accepted by many countries. The oxygen content in the near field environment of the waste container will decrease to anaerobic conditions, and hydrogen will permeation into the internal materials of container for a long time. Hydrogen evolution corrosion may cause a risk of hydrogen embrittlement. The harm of hydrogen embrittlement in metal container is far more severe than predictable uniform corrosion. It is a research hotspot that the microorganisms impact on the corrosion behavior of container materials in the deep geological environment. Microbial corrosion in deep geological environments can be divided into two types: aerobic microbial corrosion and anaerobic microbial corrosion. There is a type of hydrogen consuming microorganism in the natural environment that uses the oxidation of hydrogen as the energy for its life activities. This provides a new approach for us to study reducing the hydrogen embrittlement sensitivity of nuclear waste container materials.

## Introduction

Nuclear energy has been widely used in fields such as medicine, national defense, agriculture, and industry due to its advantages of low cost, high efficiency and no greenhouse gas generation, greatly improving human life. However, high-level nuclear waste is an inevitable waste generated during the process of nuclear energy utilization, with characteristics such as high radioactivity, long half-life and high toxicity. The various high radioactive elements pose great harm to the human body and the biosphere. For example, technetium (Tc) is a problematic fission product, as its long half-life, high fission rate and environmental mobility of high technetium salts make long-term disposal of nuclear waste more complex (Lukens and Saslow, 2017). At present, countries are vigorously developing nuclear power plants, and the rapid development of nuclear power will inevitably generate more high-level nuclear waste. For China, it is expected to produce 3,000 m^2^ high-level nuclear waste by 2030. Therefore, countries around the world have regarded efficient disposal of high-level nuclear waste as an important challenge for the development of the nuclear energy industry (Wang et al., 2004).

Regarding the disposal plan for high-level nuclear waste, researchers have proposed “deep seabed disposal,” “frozen disposal,” “hydraulic cage disposal,” “space disposal” and “deep geological disposal” (Arup, 1985; Milnes, 1985; Pang, 1989; Gao, 1995; Bradley, 1997; Shen, 2002). At present, the widely accepted solution in various countries is the deep geological disposal method, which means that high-level nuclear waste is disposed of in a suitable rock mass storage warehouse at a depth of about 500–1,000 meters from the surface, forming a “multi-barrier system” consisting of solidified high-level nuclear waste, metal container and buffer backfill materials, as well as surrounding rock and its geological environment, forming a natural barrier (SKB, 2001; Wang et al., 2005; Duquette et al., 2009). Metal container is particularly important as the first barrier for nuclear waste. Titanium and its alloys are one of the alternative materials for container due to their excellent corrosion resistance. Canada and Japan have used titanium and its alloys as alternative materials for container and have conducted extensive research (Tsujikawa and Kojima, 1990; He et al., 2002). Currently, the United States plans to use titanium droplet shields on nickel alloy containers at Yucca Mountain (Hua et al., 2005a,b, 2008; Mon, 2008; Rebak, 2009). Carbon steel has also been used as an alternative material for container in Japan, South Korea and Canada due to its advantages of simple processing and low cost (King and Padovani, 2011).

The container corrosion is closely related to the surrounding environment. The factors that affect container corrosion in deep geological environments mainly include oxygen content (partial interception of oxygen by the closed storage), temperature (decay heat release of nuclear waste), radiation (strong radioactivity of nuclear waste), microbial activity, etc. However, the near field environment of the container may change over time, for example, the oxygen content may decrease to anaerobic conditions. Winsley et al. (2011) believe that when the oxygen content around the container decreases to low oxygen concentration environmental conditions, hydrogen evolution corrosion dominates. Long term permeation of hydrogen into the interior of container materials may cause a risk of hydrogen embrittlement (HE). The good corrosion resistance of titanium and its alloys depends on the formation of a stable oxide film on their surface. If exposed to hydrogen environments, titanium is highly susceptible to hydrogen absorption. Dwivedi and Vishwakarma classified titanium as a material susceptible to HE (Dwivedi and Vishwakarma, 2018). Meanwhile, hydrogen will react with titanium and hydrides formed (Qiao et al., 2021). The titanium hydrides show brittleness. Research has shown that hydrogen ions can also be reduced and hydrogen can penetrate into carbon steel under aerobic corrosion conditions (Huang and Zhu, 2005; Tsuru et al., 2005). Therefore, hydrogen evolution corrosion will accompany the whole process of container corrosion. The harm of HE in metal container is far more severe than predictable uniform corrosion, so it is particularly important to reduce the HE sensitivity of metal container materials.

## Microbial corrosion of container

The impact of microorganisms on the corrosion behavior of container materials in the deep geological environment is currently a research hotspot. Microbial corrosion refers to the life activities of microorganisms attached to the biofilm on the surface of a material, leading to or promoting the corrosion and destruction of the material. The attachment of biofilms and the presence of metabolites alter the anodic or cathodic reaction kinetics at the metal/solution interface, leading to accelerated corrosion of metal materials (Ornek et al., 2002).

Corrosive microorganisms are often participants in the iron sulfur cycle in the environment (Huang et al., 2017). According to the types and functions of bacteria, they can be divided into sulfate reducing bacteria (SRB), sulfur oxidizing bacteria (SOB), acid producing bacteria (APB), iron oxidizing bacteria (IOB), iron reducing bacteria (IRB), nitrate reducing bacteria (NRB) and slime producing bacteria (SFB).

There are currently several main mechanisms of microbial corrosion.

(1) Cathodic depolarization theory

The cathodic depolarization theory (von Wolzogen and van der Vlugt, 1934) suggests that a “hydrogen film” will adhere to the metal surface in anaerobic environments due to the cathodic hydrogen evolution reaction, ultimately hindering the metal corrosion. This obstruction is often referred to “polarization.” SRB can be adsorbed on metal surfaces and utilize the cathodic hydrogen on the metal surface through hydrogenase in the body to reduce local hydrogen partial pressure. This “depolarization” makes metal corrosion continue. The hydrogen consumption of SRB plays a role in cathodic depolarization. The relevant reactions are as follows:

Anode reaction


(1) $$8H_{2}O\to 8H^{+}+8{OH}^{-}$$

(2) $$4Fe+8H^{+}\to 4{Fe}^{2+}+8\left[H\right]$$

Hydrogen depolarization of hydrogenase


(3) $$H_{2}{\mathrm{SO}}_{4}+8\left[H\right]\to H_{2}S+H_{2}O$$

Corrosion products


(4) $$3{Fe}^{2+}+6{OH}^{-}\to 3Fe{\left(OH\right)}_{2}$$

Total reaction


(5) $$4Fe+H_{2}{\mathrm{SO}}_{4}+2H_{2}O\to 3Fe{\left(OH\right)}_{2}+FeS$$

(2) Metabolite corrosion mechanism

Microorganisms have metabolic diversity. Therefore, the metabolites of microorganisms also have diversity, including some metabolites that can promote corrosion. For example, the metabolite H_2_S of SRB can react quickly with metal iron to generate FeS (Li et al., 2014), accelerating corrosion. The sulfide metabolites of microorganisms can also react with Fe^2+^ generated during the corrosion process to form a loose iron sulfur (FeS_x_) composite layer, which has no protective effect on metals compared to a dense FeS protective film (Liu et al., 2009). In addition, the acidic metabolites of acid producing bacteria can reduce the pH value in the environment, which is prone to serious pore corrosion and pore leakage.

(3) Concentration cell mechanism

The growth and metabolism process of microorganisms can establish various concentration batteries on metal surfaces, such as oxygen concentration batteries, metal ion concentration batteries, activation and passivation batteries, etc. The presence of concentration batteries can cause local corrosion of metals. For example, that an oxygen concentration cell with a positive potential in the rich oxygen region as the cathode and a negative potential in the poor oxygen region as the anode is formed because of the differences in oxygen consumption among different bacterial colonies within the biofilm leads to accelerated corrosion in the poor oxygen region (Abdolahi et al., 2014).

(4) Direct or indirect electronic transmission

Some microorganisms can utilize cytochrome C proteins on the surface of cell membranes and biological nanowires to directly obtain electrons from metal surfaces (Dinh et al., 2004; Reguera et al., 2005), thereby accelerating corrosion. In addition, some microorganisms can efficiently indirectly transfer extracellular electrons through reversible redox active electron mediators, For example, the phenazine like substances of Pseudomonas and the flavin like substances secreted by Shewanella (Huang et al., 2017) perform efficient indirect extracellular electron transfer, thereby accelerating metal corrosion.

**Figure 1:**
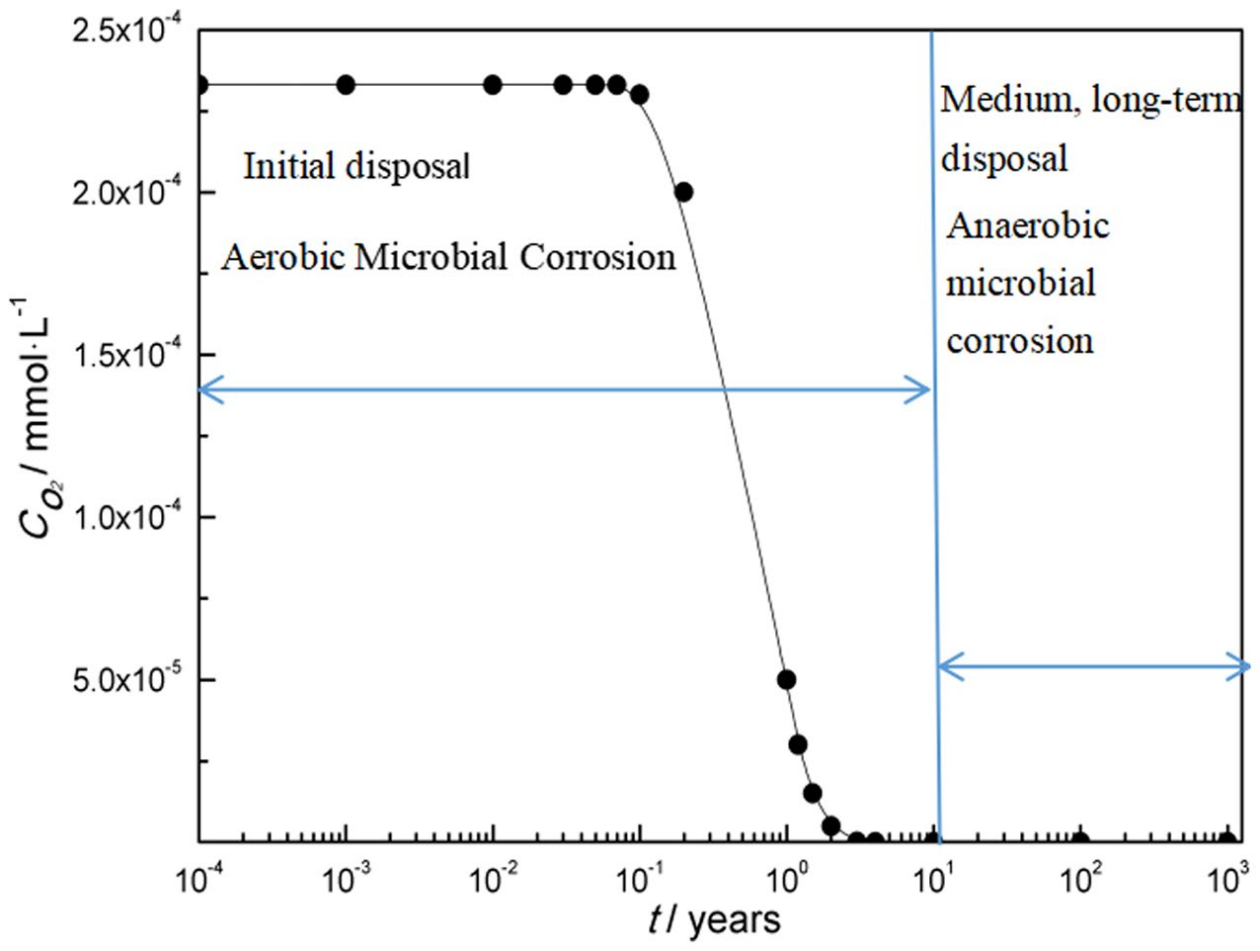
Microbial corrosion types vary with oxygen content.

Overall, there are two types of microbial corrosion in the deep geological environment of nuclear waste container. One is that in the early stages of disposal, there is a high oxygen content in the deep geological environment and aerobic microbial corrosion occurs; One is that in the medium to long term of disposal, the oxygen content decreases to an anaerobic environment, resulting in anaerobic microbial corrosion. Figure 1 shows the variation of microbial corrosion types with oxygen concentration in deep geological environments.

Aerobic conditions will certainly prevail during the repository operating phase. Many aerobic bacteria produce metabolites which can be corrosive for metallic materials in general and steels in particular (Féron and Crusset, 2014). An estimation of corrosion due to ferro-oxidizing bacteria was made on a carbon steel (Pitonzo et al., 2004) exposed to synthetic solutions representative of the waters of Yucca Mountain site (same pH, same redox and same concentration of main ions). Electrochemical measurements showed decreasing corrosion rates with time. After 1 month of testing, the mean rate of corrosion is 58 mm/year (while corrosion rate of around 30 mm/year is found on carbon steel coupons immersed in the same synthetic aqueous solutions without bacteria). Shrestha et al. examined microbial corrosion of carbon steel in synthetic bentonite pore water inoculated with natural underground water containing microorganisms over a period of 780-days under sterile and anaerobic conditions (Shrestha et al., 2021). The results indicate that nitrate-reducing bacteria could represent a potential threat to waste canisters under nuclear repository conditions.

## Hydrogen consuming microorganisms reduce hydrogen embrittlement sensitivity

**Figure 2:**
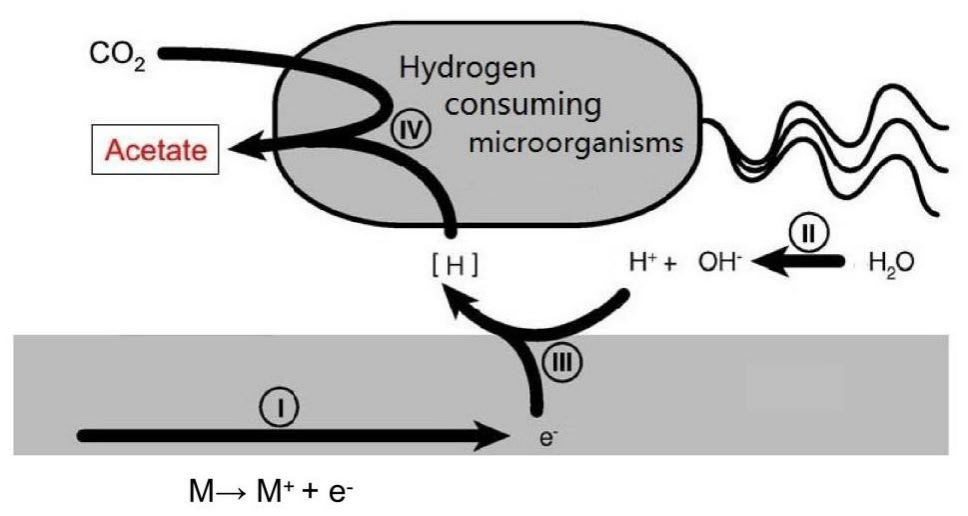
Schematic illustration of the mutual effects between hydrogen consuming organisms and metal surface.

Currently, most research is focused on the issue of microbial accelerated corrosion of nuclear waste container. However, there is little research on whether certain microorganisms exist in deep geological environments that can reduce the corrosion rate of container materials. There is a type of hydrogen consuming microorganism in the natural environment that uses hydrogen oxidation as the energy for its life activities. This provides a new approach for us to study reducing the HE sensitivity of nuclear waste container materials (Figure 2).

Collet et al. (2005) studied the effect of hydrogen consuming microorganisms on the metabolism of Clostridium thermophilum. Experiments have shown that hydrogen consuming microorganisms such as Methanothermophyter thermophilus and Moorella thermophilica increase acetic acid production by three times. Thermophilic Lactobacillus not only produces acetic acid but also by-products such as hydrogen, carbon dioxide, and lactic acid when metabolizing lactose. Thermophilic autotrophic methane bacteria play an important role as effective hydrogen removal agents, which can significantly reduce the partial pressure of hydrogen in the system and convert hydrogen into methane. Moorella thermautotrophica converts hydrogen, lactic acid, and carbon dioxide into acetic acid. Hoehler et al. (2001) involved a hydrogen consuming microbial methane producing archaea when studying the apparent minimum free energy required by microorganisms in hypoxic marine sediments. Homoacetic acid bacteria are also hydrogen consuming microorganisms, and Kotsyurbenko et al. (2001) studied their competitive behavior with methanogenic archaea for hydrogen in anaerobic environments. Lin et al. (2014) suggests that basalt aquifers on the seafloor can support hydrogen driven ecosystems. Feng et al. (2014) and Zhao (2012) conducted an experimental research on the enhancement of methane production during anaerobic digestion of sludge by adding 0 valent iron. The reason for the enhancement was that hydrogen consuming bacteria utilized hydrogen produced by iron corrosion. Mori et al. (2010) isolated 26 microbial species from petroleum facilities. The hydrogen consuming microorganisms do not have a strong promoting effect on the corrosion of iron, while the other non hydrogen consuming microorganisms cause severe corrosion of iron. These are the only literature found on hydrogen consuming microbial corrosion. Nybo et al. (2015) reviewed the use of chemotrophic autotrophic microorganisms to immobilize greenhouse gasses in the production of fuels and chemical products, which also require hydrogen consumption. There are also literature (Giblin et al., 2000; Libert et al., 2011) on the research of microbial engineering using hydrogen consuming microorganisms. It can be seen that hydrogen consuming microorganisms are widely present in nature. If the hydrogen consumption effect of these hydrogen consuming microorganisms can be utilized to consume the hydrogen generated during natural corrosion processes and reduce the diffusion of hydrogen into the metal interior, the HE possibility in metal materials can be reduced. Although microorganisms do not have a significant impact on the corrosion behavior of titanium and its alloys, if hydrogen consuming microorganisms in deep geological environments can consume excess hydrogen atoms on their surfaces, this will reduce the combination hydride of hydrogen and titanium, which is the main cause of HE in titanium and its alloys.

## Conclusion

In summary, the cathodic reaction of corrosion has shifted from oxygen absorption corrosion to hydrogen evolution corrosion due to the continuous consumption of oxygen, so there is a possibility of long-term HE in metal container during nuclear waste disposal. Unpredictable HE often has a more severe impact on nuclear waste container materials than predictable uniform corrosion. Hydrogen consuming microorganisms exist in deep geological environments, which use hydrogen oxidation as their energy for life activities. If these hydrogen consuming microorganisms can consume the hydrogen atoms generated during the natural corrosion process of the container material, thereby preventing hydrogen from permeation into the interior of the container material, greatly reducing the possibility of HE. However, most current research has focused on microbial accelerated corrosion rate of container materials in deep geological environments, only considering the adverse factors of microorganisms. However, the presence of hydrogen consuming microorganisms provides a new approach for studying the reduction of HE sensitivity for nuclear waste container materials.

## Author contributions

QZ: Writing – original draft. YJ: Funding acquisition, Supervision, Writing – review & editing. XZ: Supervision, Writing – review & editing. JD: Investigation, Supervision, Writing – review & editing. LC: Writing – review & editing. YX: Writing – review & editing.
